# Roles of Cytokines in Alzheimer’s Disease

**DOI:** 10.3390/ijms25115803

**Published:** 2024-05-26

**Authors:** Zilin Chen, Yekkuni L. Balachandran, Wai Po Chong, Kannie W. Y. Chan

**Affiliations:** 1 Department of Biomedical Engineering, City University of Hong Kong, Hong Kong, China; zilinchen9-c@my.cityu.edu.hk (Z.C.); ybalacha@cityu.edu.hk (Y.L.B.); 2 School of Chinese Medicine, Hong Kong Baptist University, Hong Kong, China; 3 Institute for Research and Continuing Education, Hong Kong Baptist University, Shenzhen 518057, China; 4 Russell H. Morgan Department of Radiology and Radiological Science, The Johns Hopkins University School of Medicine, Baltimore, MD 21205, USA; 5 Hong Kong Centre for Cerebro-Cardiovascular Health Engineering (COCHE), Hong Kong, China; 6 Shenzhen Research Institute, City University of Hong Kong Shenzhen Research Institute, Shenzhen 518057, China; 7 Tung Biomedical Sciences Centre, City University of Hong Kong, Hong Kong, China

**Keywords:** Alzheimer’s disease, neuroimmune system, cytokine, pro- and anti-inflammatory cytokines

## Abstract

The neuroimmune system is a collection of immune cells, cytokines, and the glymphatic system that plays a pivotal role in the pathogenesis and progression of Alzheimer’s disease (AD). Of particular focus are cytokines, a group of immune signaling molecules that facilitate communication among immune cells and contribute to inflammation in AD. Extensive research has shown that the dysregulated secretion of certain cytokines (IL-1β, IL-17, IL-12, IL-23, IL-6, and TNF-α) promotes neuroinflammation and exacerbates neuronal damage in AD. However, anti-inflammatory cytokines (IL-2, IL-3, IL-33, and IL-35) are also secreted during AD onset and progression, thereby preventing neuroinflammation. This review summarizes the involvement of pro- and anti-inflammatory cytokines in AD pathology and discusses their therapeutic potential.

## 1. Introduction

Alzheimer’s disease (AD), a prominent cause of dementia, leads to neuroinflammation and is especially prevalent in older adults. AD is a progressive neurodegenerative disorder that worsens with age and manifests as cognitive decline, loss of memory, disturbances in neuropsychiatric functions, and visual impairment, and it can sometimes cause executive dysfunction. The apparent neuropathological hallmarks of AD are often attributed to the extracellular accumulation of amyloid beta (Aβ) proteins, intracellular aggregation of tau proteins (neurofibrillary tangles), cerebral amyloid angiopathy, inflammation, and neurodegeneration. Over the past decade, many hypotheses have been proposed to describe AD pathogenesis, such as protopathic tau seeding, inflammation-triggered factors, cellular senescence associated with irreversible cell cycle arrest, and genetic and immune factors. The involvement of the neuroimmune system in AD progression has gained considerable attention.

**Figure 1:**
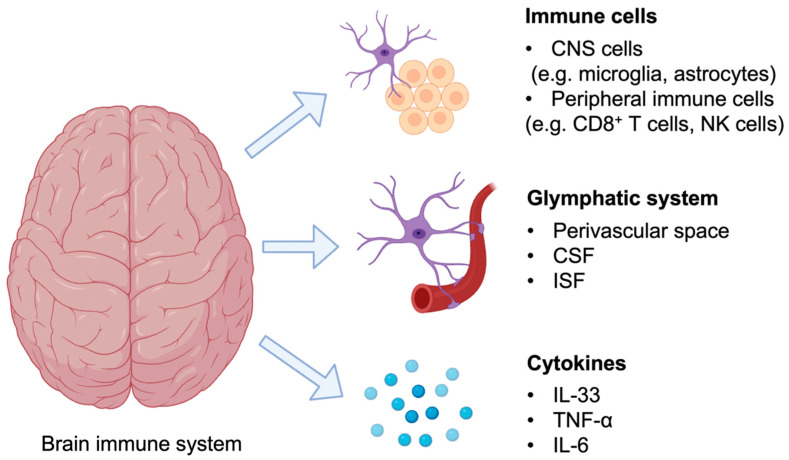
Crucial components of the neuroimmune system. The neuroimmune system comprises several crucial components: immune cells, such as resident CNS cells (e.g., microglia and astrocytes) and infiltrating peripheral immune cells (e.g., CD8+ T cells and natural killer (NK) cells); the glymphatic system, which comprises the perivascular space, CSF, and interstitial fluid; and cytokines, such as IL-33, TNF-α, and IL-6.

The neuroimmune system comprises the lymphatic–glymphatic system, resident immune cells, and cytokines (Figure 1). The glymphatic system in the brain enables solute clearance and waste removal via glia-supported perivascular channels. Recent studies have demonstrated that glymphatic system impairment alters the efficiency of perivascular cerebrospinal fluid (CSF)–interstitial fluid exchange and is often associated with AD pathogenies, leading to a significant decrease in Aβ clearance. Resident immune cells, such as microglia and astrocytes, actively participate in immune surveillance and regulate inflammation in the central nervous system (CNS) [1]. Microglia are the dominant resident immune cells in the CNS. Their proliferation, activation, and recruitment to Aβ plaque are key features of AD pathogenesis [2]. Microglia are professional phagocytes and play a critical role in Aβ plaque clearance in the AD brain [3]. Furthermore, studies have shown that astrocytes in the AD brain secrete Aβ-degrading proteases, which cleave Aβ to inhibit insoluble aggregate formation and facilitate clearance through the blood–brain barrier (BBB) [4]. However, microglia and astrocytes also aggravate inflammation in AD when activated. Microglia activation is seen with abnormal glucose metabolism starting from the early stages of AD [5,6,7], contributing to the inflammation and thereby advancing the AD progression [3]. Likewise, astrocytes in the AD brain, activated by Aβ, induce inflammation by secreting inflammasomes and release proinflammatory cytokines IL-1β and IL-18, which can contribute to innate immune defense and inflammatory diseases, including AD [1,8]. Furthermore, activated astrocytes may modulate Aβ levels through various pathways. For example, research has shown that activated astrocytes increase amyloid precursor protein (APP), β-site APP cleaving enzyme 1(BACE1), and γ-secretase, which favor Aβ aggregation. Thus, the brain’s resident immune cells are largely responsible for the inflammation observed in AD. Meanwhile, studies have also reported that peripheral immune cells, including CD8+ cytotoxic T cells, CD4+ T helper cells, and natural killer cells, infiltrate the CNS during AD development, indicating that these immune cells also play a significant role in neuroinflammation [9,10,11].

AD is associated with chronic inflammation, which inhibits neuronal function and exacerbates AD pathogenesis and progression. Cytokines, a key component of the immune system, act as a crucial mediator of neuroinflammation and play a key role in AD pathogenesis. For example, Aβ-activated astrocytes and microglia secrete various cytokines, such as tumor necrosis factor-alpha (TNF-α), interleukins (IL-1β, IL-6, IL-2, and IL-12), and interferon-gamma (IFN-γ), which promote inflammation in AD [1,12]. While anti-inflammatory cytokines, such as IL-1ra, IL-33, and IL-10, are significantly increased in the CSF and plasma in AD, reducing neuroinflammation [13]. Because of the critical role of cytokines in regulating AD pathogenesis, studies have increasingly explored interventions targeting cytokine secretion to minimize AD pathogenesis. Nevertheless, the specific roles of cytokines in AD pathology and the ways in which they interact to modulate inflammation and the immune system in AD remain unclear. A deeper understanding of the role cytokines play in the neuroimmune system, including their involvement in cellular immune responses and their regulation of AD pathology, could provide new insights into the mechanisms underlying AD and potential therapeutic approaches.

This review provides an overview of proinflammatory cytokines including IL-1β, IL-6, IL-12, IL-23, IL-17, and TNF-α, which exert detrimental effects in AD, as well as anti-inflammatory cytokines IL-2, IL-3, IL-33, and IL-35. The immunomodulatory functions of these cytokines may provide insights into AD pathogenesis and potential therapeutic approaches. We further discuss the complex interactions between cytokines and highlight their therapeutic potential regarding AD pathologies.

## 2. Cytokines in the Pathogenesis of AD

Cytokines are secreted proteins that play a vital role in the initiation, maintenance, and regulation of the immune response [14]. Additionally, cytokines serve as important mediators of cell-to-cell communication. They enable long-range intercellular communication, allowing for the global regulation of inflammation rather than just localized effects [15]. The onset and pathogenesis of AD entail the modulation of multiple cytokines that play roles in inflammation severity; conversely, the release of anti-inflammatory cytokines can mitigate inflammatory response. The following section discusses the role of pro- and anti-inflammatory cytokines in AD pathogenesis.

### 2.1. Proinflammatory Cytokines

Proinflammatory cytokines are a group of signaling molecules primarily produced by immune cells that promote inflammation and contribute to the immune response. The well-known proinflammatory cytokines IL-1β, IL-6, IL-12, IL-23, IL-17, and TNFα have been implicated in the progression of various diseases, including type I diabetes, multiple sclerosis (MS), uveitis, and atherosclerosis [16]. Notably, these cytokines also play an immunoregulatory role in AD [17,18,19,20,21].

#### 2.1.1. IL-1β

IL-1β is a potent proinflammatory cytokine primarily secreted by myeloid cells. The maturation of IL-1β occurs via the proteolytic cleavage of pro-IL-1β by the inflammasome-activated caspase-1 protein, and mature IL-1β is trafficked to the plasma membrane and secreted extracellularly as a cytokine [22]. IL-1β binds to the membrane receptor IL-1R1 and recruits the IL-1R3 co-receptor to promote local inflammation and activate proinflammatory myeloid cells, including neutrophils, monocytes, and macrophages [23]. Furthermore, IL-1β activates several type-3 immune cells, such as IL-17-producing lymphoid cells, Th17 cells, and Tc17 cells, which play pathogenic roles in various inflammatory and autoimmune diseases [24]. IL-1β can also interact with IL-23 to promote IL-17 production. Indirectly, IL-1β supports the differentiation of IL-17-producing Th17 cells by inducing innate immune cells to produce IL-6 [23].

In the brain, IL-1β secretion contributes to neuroinflammation by activating the microglial cells to produce neurotoxic substances, thereby promoting neuronal death [25]. Moreover, IL-1β exacerbates neurodegeneration by recruiting and activating other immune cells, such as monocytes and leukocytes [25]. The upregulation of the proinflammatory cytokine IL-1β has been reported as an early indicator of various neuropathogenic diseases, including AD [25,26,27]. Aβ stimulates the secretion of IL-1β from microglia during AD onset [26,27]. As AD progresses, microglia are activated and cluster around Aβ plaques to promote their phagocytosis. Concurrently, these microglia also contribute to increased levels of IL-1β, thereby becoming a primary cellular source of this proinflammatory cytokine in the AD brain [25]. The secreted IL-1β further amplifies the neuroinflammation in AD by inducing other cytokines and chemokines, such as IL-6 and IL-17, leading to a cycle that exacerbates neuronal damage, cell death, and neurodegeneration [23,25]. Increased plasma IL-1β levels are observed in patients with AD, suggesting that systemic chronic inflammation is associated with the development of AD pathogenesis. In addition to IL-1β, other cytokines from IL-1 family also demonstrate potential in indicating AD progression. Elevated levels of IL-1Ra, IL-33, IL-18BP, and soluble receptors sIL-1R1, sIL-1R3, and sIL-1R4 have been observed in AD patients [18]. Interestingly, free IL-18 and sIL-1R2 show differential expression between mild cognitive impairment (MCI) and AD [18], indicating their potential utility as markers for assessing the progression from MCI to AD.

#### 2.1.2. IL-17

IL-17′s involvement in inflammatory pathology has been extensively studied. CD4+ and CD8+ T cells largely contribute to IL-17 production. Furthermore, as a proinflammatory cytokine, IL-17 is also secreted by innate immune cells, including mast cells, natural killer cells, and innate lymphoid cells. Generally, IL-17 production is driven by inflammatory cytokines, particularly IL-1β, IL-6, and IL-23 [28]. Although IL-17 plays a protective role in host immunity against pathogens, it can also act as a pathogenic cytokine in chronic neuroinflammatory diseases, such as MS and uveitis [28,29]. Therefore, it is important to consider the delicate balance between the pathogenic and protective roles of IL-17 when exploring therapeutic approaches targeting IL-17 and its related signaling pathways. However, in neuroinflammation, IL-17 predominantly acts as a pathogenic cytokine [24]. In neuroinflammation, IL-17 contributes to disrupting the BBB and encourages inflammation in the CNS [30]. Furthermore, a recent study demonstrated that IL-17 secreted by Th17 was involved in neuron degeneration in Lewy body dementia [31]. In AD, Th17 cells and their signature cytokine IL-17 play a major role in disease onset and progression. They are observed extensively in the AD brain, suggesting that they play a pathogenic role in disease progression [21]. Furthermore, IL-17 has been reported to modulate Aβ metabolism. IL-17 upregulates APP and BACE1 expression, thereby promoting Aβ production and impairing Aβ clearance by inhibiting microglial phagocytosis. Aβ stimulates myeloid immune cells through Toll-like receptors and activates the NF-κB signaling pathway, leading to the production of IL-17. This creates a positive feedback loop involving Aβ accumulation and IL-17 induction [21]. Thus, cellular and animal studies have explored blocking IL-17 signaling as a therapeutic approach to ameliorate Aβ-induced neuroinflammation and memory impairment as well as attenuate cognitive deficits [32,33].

#### 2.1.3. IL-12 Family

IL-12 is a heterodimeric cytokine family comprising four members: IL-12, IL-23, IL-27, and IL-35. The major cytokines of the IL-12 family consist of α-chain (IL12p35, IL23p19, and IL27p28) and β-chain (p40 and Ebi3) subunits and are primarily secreted by dendritic cells, monocytes, and macrophages [34]. IL-12 promotes the differentiation of IFN-γ-producing Th1 cells involved in tissue inflammation, and IL-23 plays a key role in driving Th17 cells by stabilizing the Th17 phenotype for the continued expression of cytokines [35]. IL-12 family cytokines signal through the JAK–STAT pathway to initiate the downstream production of proinflammatory molecules [35]. IL-12 and IL-23 are involved in inflammatory and autoimmune responses in various diseases, including inflammatory bowel disease, systemic lupus erythematosus, rheumatoid arthritis, and MS [34,36]. In the early stages of AD, elevated IL-12 and IL-23 secretions are correlated with cognitive performance [19,37,38]. During the onset of AD, activated microglia upregulate the secretion of IL-12 and IL-23, transforming the microglia to a pathogenic state with an impaired Aβ clearance function [19,39]. IL-12 members also modulate immune cells to secrete other cytokines, increasing AD severity. For example, IL-23 induces Th17 cell differentiation to produce IL-17. In addition, IL-23 contributes to Aβ accumulation and impairs the microglial, affecting Aβ clearance [32]. Targeting the common β-chain p40 of IL-12/IL-23 and inhibiting IL-12/IL-23 signaling can reduce pathogenic microglia, decrease Aβ accumulation, and reverse the memory defects in an AD animal model [19]. Furthermore, blocking IL-23 reverses the impaired clearance function of microglia even in the presence of IL-17, suggesting that IL-23 plays a distinct role from IL-17 in AD development [32]. The involvement of IL-12 and IL-23 in AD progression indicates their therapeutic potential in lessening disease severity.

#### 2.1.4. IL-6

The structure of IL-6 is characterized by a motif comprising four α-helix bundles. IL-6 is recognized by the IL-6 receptor, which has two subunits, IL-6R and gp130, forming a heterodimer [40]. The binding of IL-6 to IL-6 receptors initiates the JAK–STAT signaling pathway. IL-6 expression is strictly controlled through transcription and locally secreted in response to tissue inflammation or infection. IL-6 is found to be associated with pathological conditions characterized by chronic inflammation and autoimmunity, including AD [41]. Elevated IL-6 levels have been reported in both brain and plasma of AD patients. This increased IL-6 expression is positively correlated with impaired cognitive performance and pathological changes in the AD brain, and such changes are often recognized by T2 hyperintense spots on magnetic resonance imaging (MRI) [42]. Genetically, overexpression of the IL-6 gene has been observed in patients diagnosed with late AD [20,43], suggesting that IL-6 impacts AD progression and severity. Furthermore, individuals carrying the G allele in the IL-6-174C/G polymorphism are more susceptible to AD [44]. Taken together, these studies highlight the pathogenic role of IL-6 in AD; thus, modulating the expression of IL-6 has been explored as a therapeutic approach. The overexpression of IL-6 and its downstream signaling molecule STAT3 caused memory impairment in mice [42], and inhibiting either of them reversed the impairment and restored the animal’s memory. Additionally, downregulating IL-6 expression alleviates glucose intolerance [42], a common dysfunction exaggerated by IL-6 in AD progression [45]. This suggests that IL-6 may contribute to AD progression by disrupting glucose metabolism. Upon binding to its receptor, IL-6 triggers the activation of PI3K/Akt signaling pathway, resulting in mTOR activation and PTEN inhibition. Activated mTOR can modulate the functioning of insulin receptor substrate 1 (IRS1), and consequently lead to impaired insulin sensitivity and glucose uptake. Simultaneously, the inhibition of PTEN also contribute to these negative effects on insulin sensitivity and glucose uptake [46]. Furthermore, the β-secretase BACE1 directly cleaves the IL-6 receptor gp130, suggesting that IL-6 could be a potential therapeutic target for regulating the Aβ burden [47]. Meanwhile, IL-6 upregulation is also associated with poor prognosis in other diseases, including cancers, and COVID-19 [48], reflecting its proinflammatory role in the immune system. Additionally, IL-6 is one of the major markers for cellular senescence [49], which indicate the potential development of age-related diseases, chronic inflammation and tissue dysfunction. Therefore, it is crucial to conduct more research to determine the exact involvement of IL-6 in AD and its potential as a therapeutic target.

#### 2.1.5. TNF-α

TNF-α is a homotrimer and proinflammatory cytokine generally secreted by activated macrophages and lymphocytes [50]. The involvement of TNF-α has been observed in various autoimmune diseases, such as uveitis, rheumatoid arthritis, MS, and inflammatory bowel disease [51]. Generally, the soluble form of TNF-α facilitates various inflammatory activities via the TNF type 1 receptor (TNFR1) and TNF type 2 receptor (TNFR2). Soluble TNF-α is derived when TNF-α-converting enzyme cleaves transmembrane TNF-α expressed on activated macrophages or T cells. The binding of TNF-α to TNFR1 and TNFR2 triggers the NF-κB/MAPK signaling pathway, inducing inflammation, tissue degeneration, cell proliferation, and survival. Additionally, TNFR1 induces apoptosis via the caspase-8 signaling pathway and activates MLKL to induce necroptosis [51]. TNF-α is implicated in various neuropathological processes, including AD pathogenesis. The activation of the TNF-α/TNFR1 signaling pathway promotes neuronal necroptosis, which greatly contributes to the development of AD [52]. TNF-α induces the rapid exocytosis of AMPA receptors, boosts glutamatergic transmission, and suppresses long-term potentiation, inhibiting learning and memory [53]. TNF-α affects microglia in AD. In the brain, TNF-α modulates the functionality of microglia by transforming into a pathogenic form characterized by dysregulated Aβ clearance. TNF-α’s impairment of microglial activity becomes more severe with age; TNF-α alters synaptic transmission without altering Aβ metabolism in young AD animal model [53], whereas it causes increased Aβ production and reduced microglial clearance in adult AD animal model [54]. Peripherally, TNF-α overexpression activates microglial activation, which is linked to leukocytes, perivascular macrophage infiltration, and synaptic degeneration [55]. Further investigation is needed to elucidate how the peripheral TNF-α signal reaches the brain, as this understanding may help reveal the relationship between peripheral inflammatory diseases and AD.

Regarding genetics, TNF-α-850 polymorphism is associated with AD risk [17]. The G308A polymorphism found among the Chinese population leads to an increased risk of AD in this population than in European populations [56]. Although TNF-α may exhibit genetic variations among populations, there is consistent evidence suggesting an upregulation of TNF-α expression in both brain and serum in AD pathogenesis [57,58,59]. Collectively, the literature indicates that TNF-α significantly exacerbates AD progression. Therefore, targeting TNF-α is considered a valid therapeutic approach to mitigate AD severity. Various TNF-α blocking agents (including etanercept, infliximab, adalimumab, and Theracurmin) have demonstrated the ability to reduce microgliosis, neuronal loss, tau tangles, and Aβ accumulation. Furthermore, both pre-clinical and clinical studies have indicated their potential in improving cognitive function [60,61,62].

### 2.2. Anti-Inflammatory Cytokines in AD

In addition to the detrimental cytokines, a group of cytokines possess anti-inflammatory properties in AD. Anti-inflammatory cytokines, such as IL-2, IL-3, IL-33, and IL-35, play crucial roles in mitigating inflammation and hold therapeutic potential for AD.

#### 2.2.1. IL-3

IL-3 is a cytokine mainly produced by activated T cells that stimulate the proliferation of other immune cells. It is a multi-lineage colony-stimulating factor that prevents cell death and stimulates the growth of immature progenitor cells. IL-3 is a multifunctional cytokine and plays a pivotal role in inflammatory and autoimmune diseases. IL-3 contributes to several chronic inflammatory diseases, such as sepsis [63], MS [64], lupus nephritis [65], arthritis [66], and AD [67], and promotes anti-inflammatory activity to ease inflammation. In AD, the expression level of the IL-3 receptor IL-3RA is elevated and is positively correlated with Aβ40 and Aβ42 accumulation [67]. Furthermore, the expression of IL-3 in the CSF is associated with core AD biomarkers, such as Aβ42, p-tau, and t-tau proteins at baseline [68]. In AD, IL-3 deficiency leads to Aβ accumulation and causes cognitive impairment. Notably, in mice without disease, IL-3 deficiency does not impact BBB permeability, neurogenesis, neuronal death, microglia activation and proliferation [67], suggesting the specific role of IL-3 toward AD. Regarding the mechanism, IL-3 modulates microglia activity by catalyzing the microglial clearance mechanism. Upon recognition of Aβ in the AD brain, the microglia upregulate the expression of the IL-3Rα receptor and binds to the astrocyte-derived IL-3, and such interaction activates the microglia. The IL-3-activated microglia exhibit acute immune response, increase in motility, and capability to cluster and clear the Aβ and tau aggregates in AD pathology [67].

#### 2.2.2. IL-35

IL-35, the most recently added member of the heterodimer IL-12 cytokine family, comprises two subunits, Ebi3 and p35, which bind to the receptors gp130 and IL-12Rβ2. Unlike other IL-12 family members (IL-12 and IL-23, secreted by antigen-presenting cells), IL-35 is mainly produced by regulatory B cells (Bregs) and regulatory T cells (Tregs) [69]. These IL-35-producing Bregs and Tregs exert potential anti-inflammatory effects by inhibiting the production of Th1 and Th17 cells and increasing the activity of Treg cells and IL-10-producing Breg cells [70]. IL-35 suppresses inflammation, autoimmune diseases, and neurodegenerative diseases, including MS and AD [69,71]. Furthermore, IL-35 expression and IL-35-producing Treg and Breg cells are positively correlated with AD pathogenesis [71,72], suggesting that IL-35 plays a compensatory role in response to AD. Although IL-35-producing Tregs protect against AD progression [73], no direct evidence has demonstrated that AD amelioration is dependent on IL-35. Breg cells are upregulated in early AD and play a protective role in AD via IL-35. These IL-35-producing Breg cells reduce Aβ load and cognitive impairment in the 5xFAD mouse model. In terms of the mechanism, IL-35 inhibits BACE1 transcription in neurons via the SOCS1/STAT1 signaling pathway [71], supporting its potential therapeutic role in AD.

#### 2.2.3. IL-2

IL-2 is a four-α-helix cytokine that binds to the receptors IL-2Rα (CD25), IL-2Rβ (CD122), and γc (CD132). IL-2 triggers intracellular pathways, such as JAK–STAT, PI3K, and MAPK [74,75] to modulate the inflammatory response. During the immune response, IL-2 has dual and opposing functions. It promotes Treg generation, survival, and activity; simultaneously, it plays a prominent role in promoting the generation and proliferation of effector T cells. Regarding its therapeutic potential, IL-2 exposure activates Tregs and controls the development of autoimmune disease. However, minimizing the compromise of expanding effector cells during treatment remains a significant challenge [75]. Generally, IL-2 expression in AD is downregulated in the hippocampus [76]. Peripheral IL-2 injection induced IL-2 expression and increased the Treg population in the brain. IL-2 therapy also rescued memory impairment and restored spinal density [76], lessening the amyloid load and plaque deposition in AD mice. During dysfunction, IL-2 recruits astrocytes around Aβ plaques and activates them via the JAK/STAT3 pathway [76]. The activated astrocytes protect the neurons by forming a physical barrier around plaques [76], reducing Aβ deposition by degrading [77] and internalizing the protein [78].

#### 2.2.4. IL-33

IL-33, generally recognized as an alarmin, is a damage-associated protein of the IL-1 family secreted by non-hematopoietic cell types, such as endothelial and epithelial cells, fibroblasts, and brain cells, during cellular damage or necrosis. IL-33 exhibits both intracellular and extracellular localization, and demonstrates distinct functional activities depending on its location. For example, in the nucleus, IL-33 acts as a transcriptional regulator and joins with chromatin, acting as an endogenous alarmin to regulate the transcriptional system and maintain immune homeostasis [79]. However, when released into the extracellular space, IL-33 interacts with the receptor ST2 to initiate multiple downstream signaling pathways, such as the MAPK and NF-κB pathways. The IL-33 functional ligand ST2 exists in transmembrane (ST2L) and soluble (sST2) forms. ST2L is bound to the cell membranes of lymphoid cells and APCs; sST2 functions as a decoy receptor in biological fluids, limiting the interaction between IL-33 and ST2L [80]. The binding of IL-33 to the receptor ST2L leads to MYD88 recruitment and kinase activation and triggers the MAPK and NF-κB pathways, resulting in cell proliferation, survival, and cytokine secretion [81,82]. IL-33 possesses a pleiotropic immune regulatory function in the disease context. For example, nuclear IL-33 is a pro-pathogenic factor that contributes to disease progression in skin cancer, pancreatitis, pancreatitis-associated PDAC [83], and hepatocellular carcinoma [84]. Nevertheless, IL-33 promotes Treg expansion in the liver, which is promising for CMV-induced hepatitis treatment.

IL-33 cytokine and its functional receptor ST2 are observed at higher levels in pre-clinical animal models and patients with AD [3,18,85,86,87]. As an alarmin, IL-33 potentially influences the Aβ accumulation and could be a promising candidate for AD treatment. Upregulating the cellular expression of IL-33 reduces Aβ secretion [88] and restrains GSK-3β-mediated tau phosphorylation, thereby decreasing tau tangles [89]. Furthermore, the exogenous administration of IL-33 prevented Aβ accumulation by promoting the phagocytic activity of microglia via IL-33/ST2 signaling and the PU.1-dependent transcriptional pathway in APP/PS1 mice [3,90]. IL-33 influences monocyte polarization to the M2 anti-inflammatory phenotype, leading to a decreased production of proinflammatory cytokines, such as IL-1β and IL-6 [91], which could reduce dysfunction in AD. Genetic phenotyping in some patients with AD has demonstrated a downregulated IL-33 expression [88,92,93], evidencing the protective role of IL-33 in AD pathogenesis [94].

## 3. Interaction between Protective and Detrimental Cytokines in AD

**Figure 2:**
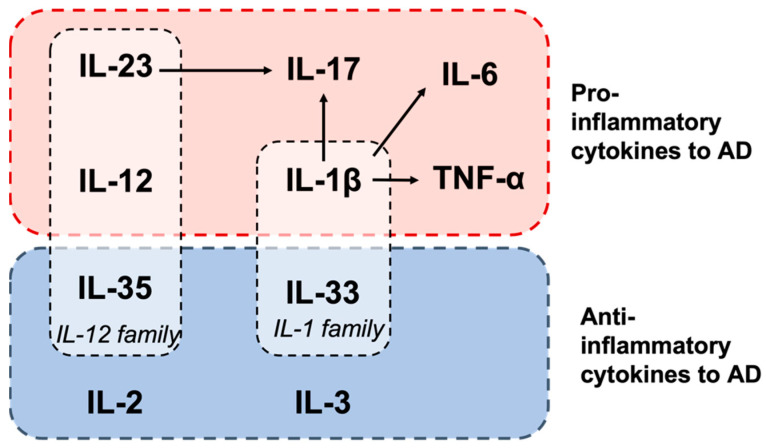
Interaction of cytokines involved in AD pathology. Proinflammatory cytokines (red box) and anti-inflammatory cytokines (blue box) in AD. Arrows indicate the induced effects.

Because AD onset and progression are associated with many pro- and anti-inflammatory cytokines, it is essential to discuss the interactions between cytokines when exploring the function of a single cytokine. Cytokines can directly modulate inflammation by triggering or inhibiting inflammatory processes; they also impact other cells and cytokines, influencing overall immune response regulation [14]. Interactions between specific cytokines can contribute to the maintenance and expansion of particular immune cell populations; for example, the AD proinflammatory cytokines IL-1β, IL-23, and IL-17. IL-1β promote the differentiation of naive T cells into Th17 cells [23], and IL-23 sustains and expands the Th17 population [35]. This expansion is facilitated by a positive feedback loop that upregulates the expression of IL-1β and IL-17 [28], further promoting Th17 cell differentiation and function (Figure 2). The involvement of these cytokines in AD pathology suggests that Th17 cells play a role in AD progression and that these cytokines may promote AD pathology through a shared pathway. It is noteworthy that the sustained expression of proinflammatory cytokines in AD may be associated with the senescence-associated secretory phenotype (SASP), a state in which cells secrete proinflammatory molecules during cellular senescence. Since aging is one of the primary risk factors for dementia, cellular senescence has been proposed as a potential mechanism toward AD [95]. Several studies have reported the accumulation of senescent cells in the AD brain [49,96]. Concurrently, tau aggregation and Aβ deposition have been shown to induce cellular senescence [97], indicating a strong association between senescence and AD pathophysiology. However, it is important to note that cellular senescence occurs not only in AD, but also during normal aging and chronic inflammation such as diabetes. Consequently, determining whether the dysregulation of cytokines is a direct result of SASP and whether this contributes to the development of AD necessitates further investigation.

Cytokines belonging to the same family can exhibit varying characteristics in the context of a disease. For instance, although IL-35 belongs to the same IL-12 family as IL-12 and IL-23 [69], they play conflicting roles in AD. IL-35 potentially protects against AD by mitigating the Aβ load and cognitive impairment, whereas IL-12 and IL-23 contribute to Aβ accumulation and exacerbate AD. Similarly, IL-33 and IL-1β are members of the IL-1 family [79], yet they display opposing effects on AD progression. In most cases, cytokines within the same family have similar functions because of their structural and functional homology. However, variations in function between cytokines can arise because of the combination of distinct receptors, which may influence the signaling pathway in different ways, thereby causing variation in immune regulation. Moreover, the differential expression of cytokines in various cell types and tissue environments can influence their role in inflammation. IL-33′s deviation from the proinflammatory role of IL-1β could be due to its specific interaction with the ST2 receptor, which triggers a distinct signaling pathway. Overall, the relationships and interactions between cytokines highlight the complexity of cytokine singling. Understanding these differences can provide valuable insights into AD pathology and the development of cytokine-modulated therapies.

## 4. Cytokine Therapeutic Targets

**Figure 3:**
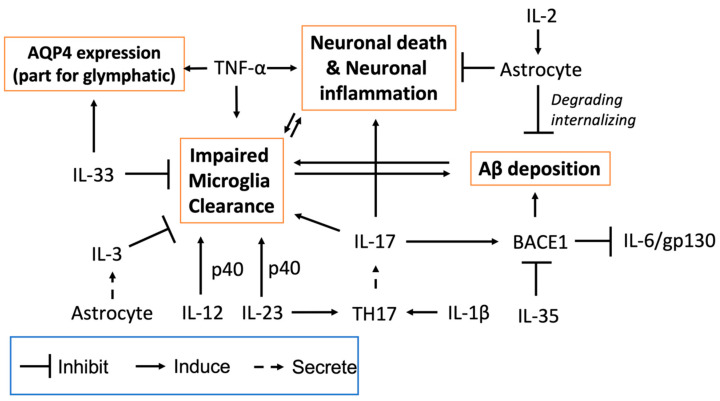
Correlation between cytokines and AD pathologies. Cytokines and their interaction between AD pathologies, such as neuronal death and neuronal inflammation, impaired microglia clearance, and Aβ deposition.

Several studies have explored the potential of cytokines as therapeutic targets for AD. Although the precise pathogenesis of AD is still not fully understood, several pathological dysfunctions have been identified as significant contributors, including Aβ deposition, impaired microglial clearance, glymphatic clearance dysfunction, and neuronal inflammation and impairment. These disorders are interconnected with each other. Cytokines play a crucial role in regulating inflammation, and many of them can be modulated therapeutically to suppress neuroinflammation by inhibiting proinflammatory cytokines or promoting the expression of anti-inflammatory cytokines. Therefore, targeting cytokines can mitigate the inflammatory response in AD and address its underlying disorders (Figure 3).

When targeting the AD symptoms, cytokines exhibit therapeutic potentials. The abnormal accumulation of tau tangles and Aβ plaques is widely recognized as a biomarker for AD and is thus considered a therapeutic target. Aβ clearance can occur through the glymphatic system and microglia-mediated degradation. The glymphatic system is a fluid clearance pathway that flows directionally through the brain, with influx from para-arterial regions and efflux to the peripheral system. In this process, metabolic waste is non-selectively cleared from the interstitial space, highlighting its potential significance as a therapeutic target for AD [98]. It has been reported that IL-33 can enhance AQP4 expression on astrocytes and promote the drainage of abnormal tau from the brain via the ventricular fluid [99]. Although direct evidence linking cytokines to glymphatic function in AD is limited, the studies above suggest that targeting cytokines could be a potential strategy for improving glymphatic function.

BACE1 plays a major role in generating Aβ peptides, and Aβ is one of its cleavage products. IL-35, an anti-inflammatory cytokine, can inhibit BACE1 transcription, thereby reducing Aβ accumulation [71]. Conversely, IL-17 reportedly induces the expression of BACE1; in turn, BACE1 is necessary for IL-17A production [100,101]. Consequently, the administration of anti-IL-17A antibodies has been investigated as a potential approach to mitigate the Aβ burden in AD [32,33]. However, IL-17A is involved in multiple neuroinflammatory conditions, such as MS and stroke [24,28], which share common IL-17 pathways during inflammation; this raises questions about the specificity of IL-17 inhibition as a treatment approach for AD. As discussed above, the pathological characteristics of AD, such as neuronal death, impaired microglial clearance, and glymphatic clearance, are also observed in other neurodegenerative disorders [102,103,104], suggesting that cytokine targeting may not be specific to AD but provide a broad therapeutic approach for multiple neurodegenerative diseases. Moreover, some cytokine dysregulation is not unique to AD and is observed in other disorders. Further investigation is needed to determine the extent of the role cytokines play in AD pathogenesis. Gaining a better understanding of the immunoregulation of cytokines in AD may aid in developing more specific therapeutic strategies for the disease.

Furthermore, anti-cytokine drugs have been proposed to modulate the inflammatory response in AD. Drugs such as etanercept, infliximab, and adalimumab are used to target TNF-α, binding to it and neutralizing its effects, reducing AD risk and preserving cognition [60]. Cytokines in IL-1 family is another therapeutic target to AD. Anakinra acts as a IL-1β receptor antagonist, reducing neuroinflammation, tau phosphorylation, cognitive deficits and synaptic loss [105], while canakinumab directly neutralizes IL-1β, preventing its proinflammatory actions [105]. IL-6 is a proinflammatory cytokine that plays a role in the immune response and neuroinflammation, and tocilizumab blocks the IL-6 receptor, preventing IL-6 from binding and signaling, resulting in learning and spatial memory improvement and Aβ reduction [106]. Ustekinumab inhibits the p40 subunit of IL-12 and IL-23 [107], both of which exhibit their amelioration capacity to AD mice [19]. This suggests that ustekinumab has the potential to be an effective therapy for AD. However, despite its promising results in mice, ustekinumab has not yet been used in clinical practice. There are several factors that should be considered when evaluating the potential use of anti-cytokine drugs for AD. Firstly, translating pre-clinical studies to clinical applications is challenging. Despite the positive outcomes in animal models, researchers still face the significant hurdle of determining the appropriate dosage to be administered that ensures the safety and efficacy for human subjects. Additionally, the cost is a concern that could hinder its accessibility and affordability for patients. Furthermore, long-term use may lead to the accumulation of side effects and drug resistance over time. Moreover, the intricate balance of immune homeostasis should be taken into account when considering the use of anti-cytokine drugs. Cytokines play vital roles in regulating the immune system, and any alterations in their levels can trigger cascading reactions and result in unpredictable consequences. Therefore, further investigation is necessary to balance the control of AD with maintaining immune homeostasis.

## 5. Conclusions

This review summarized the intricate neuroimmune system within the brain, highlighting the involvement of various cells and cytokines in the immune response, specifically in AD. The interaction between these cells and cytokine signaling pathways is complex. We specifically addressed the cytokines implicated in AD pathogenesis and progression, categorizing them according to their regulatory roles as proinflammatory or anti-inflammatory. The dysregulation of these cytokines in AD highlights their significance in the disease’s development and suggests that they represent potential diagnostic markers and therapeutic targets. Furthermore, we summarized the intricate interactions among cytokines, including their shared signaling pathways and ability to modulate each other’s expression. Finally, we discussed the relationship between cytokines and neuronal health, microglial clearance, glymphatic function, and Aβ deposition, which are hallmarks of AD. Deep investigations into the mechanisms by which cytokines regulate neuroimmune responses are warranted to develop targeted therapeutic approaches for AD.

## Data Availability

Not applicable.
